# Single-Molecule Detection in Nanogap-Embedded Plasmonic Gratings

**DOI:** 10.5772/61094

**Published:** 2015-01-01

**Authors:** Biyan Chen, Avinash Pathak, Keshab Gangopadhyay, Peter V. Cornish, Shubhra Gangopadhyay

**Affiliations:** 1 Department of Electrical and Computer Engineering, 139 and 141A Engineering Building West, University of Missouri, Columbia, MO, USA; 2 Nanos Technologies LLC, Business Incubator Center, Columbia, MO, USA; 3 Department of Biochemistry, 117 Schweitzer Hall, University of Missouri, Columbia, MO, USA

**Keywords:** single-molecule detection, DNA/RNA duplex, plasmonic gratings, nanogaps, epifluorescence microscope

## Abstract

We introduce nanogap-embedded silver plasmonic gratings for single-molecule (SM) visualization using an epifluorescence microscope. This silver plasmonic platform was fabricated by a cost-effective nano-imprint lithography technique, using an HD DVD template. DNA/ RNA duplex molecules tagged with Cy3/Cy5 fluorophores were immobilized on SiO_2_-capped silver gratings. Light was coupled to the gratings at particular wavelengths and incident angles to form surface plasmons. The SM fluorescence intensity of the fluorophores at the nanogaps showed approximately a 100-fold mean enhancement with respect to the fluorophores observed on quartz slides using an epifluorescence microscope. This high level of enhancement was due to the concentration of surface plasmons at the nanogaps. When nanogaps imaged with epifluorescence mode were compared to quartz imaged using total internal reflection fluorescence (TIRF) microscopy, more than a 30-fold mean enhancement was obtained. Due to the SM fluorescence enhancement of plasmonic gratings and the correspondingly high emission intensity, the required laser power can be reduced, resulting in a prolonged detection time prior to photobleaching. This simple platform was able to perform SM studies with a low-cost epifluorescence apparatus, instead of the more expensive TIRF or confocal microscopes, which would enable SM analysis to take place in most scientific laboratories.

## 1. Introduction

Single-molecule (SM) detection techniques are revolutionizing biological inquiries from ensembles of molecules to individual molecules in both life sciences and materials science [1–4]. Recent advances in SM measurements with unprecedented precision and clarity have enabled the observation of conformational states and reaction dynamics at the molecular level, in real time [3–6]. The most widely used approaches for SM experiments are epifluorescence [7], confocal [8] and total internal reflection fluorescence microscopy (TIRFM) [9,10]. TIRFM relies on the use of an evanescent field generated by total internal reflection of incident laser radiation to image the region within a ∼100 nm vicinity of the platform. Epifluorescence uses free space radiation to illuminate the entire bulk of the platform on which the SM measurement is performed. As a consequence, TIRFM is able to provide far better signal-to-noise ratio (SNR), albeit at the cost of requiring an expensive apparatus to create the evanescent field [7]. Confocal microscopy has the highest resolution of the listed methods, and the ability to focus on any given plane and focal point, but it requires expensive equipment [11]. Conventional SM imaging techniques rely heavily on TIRFM or confocal microscopy, due to the high background noise obtained with epifluorescence imaging, which drives up equipment costs.

Improving the detection capabilities of SM platforms requires a two-fold approach: improving the intensity of fluorescent radiation from the individual molecules, and increasing SNR. One method recently proposed is the application of the principles of grating-based surface plasmon resonance (SPR) [12] to enhance the intensity of fluorescence from the molecules under observation [6, 13–15]. SPR generates a highly concentrated evanescent electric (E-) field at the metal-dielectric interface region, significantly increasing the fluorescence excitation and emission intensity of the fluorophores immobilized near the plasmonic surface [16–20]. Fluorescence can also be enhanced by localized surface plasmon resonance (LSPR) via the concentration of coupled light, using nanostructured singularities such as nanoantennae [5,21] and nanoresonators [15]. Our previous work shows that the integration of plasmonic gratings and random nanogaps can provide a fluorescence enhancement in excess of 100-fold, with respect to that of glass [22]. Such a high level of fluorescence enhancement is promising for the prospect of achieving SM detection using less complex and expensive epifluorescence microscopes.

In this study, we utilized a cost-effective nanogap-embedded silver plasmonic grating, fabricated by a nano-imprint lithography technique from a store-bought HD DVD [22], to perform SM studies on a DNA/RNA hybrid duplex tagged with Cyanine 3 (Cy3) and Cyanine 5 (Cy5) fluorophores. An additional 10 nm SiO_2_ capping layer, deposited on the silver gratings, was used as a spacer layer to avoid fluorescence quenching by the metal, to reduce silver degradation in the air or in an aqueous environment, and to enable surface bonding with hydroxyl to increase the adsorption of biotinylated BSA. The intensity from the fluorophores on our platform was compared to that of fluorophores on quartz slides observed under TIRFM, which is the *de facto* platform for SM fluorescence studies. The nanogaps form randomly within the plasmonic grating cross-section and act as “lighting rods”, concentrating the electromagnetic radiation and enhancing fluorescence emission in comparison to plain quartz slides. Furthermore, the gratings with nanogaps were able to concentrate light sufficiently for one to perform SM studies by utilizing a low-cost epifluorescence microscope as a substitute for the more costly TIRFM or confocal setup. Given the low cost of the platform, and the comparative ease with which the silver gratings can be used for SM studies, it is envisioned that its use will help bring SM analysis capability to most scientific laboratories.

## 2. Methods

### 2.1 Sample Preparation for Single-molecule Experiments

The silver grating platforms were fabricated by a nanolithography process using HD DVD grating templates, as described previously [22–24]. Briefly, poly(methylsilses-quioxane) (PMSSQ) with transferred grating structures was first stamped onto a silicon wafer. The mechanical stresses generated during the stamping process induced the formation of random nanogaps with different sizes and distribution. Subsequently, a 2 nm titanium layer acting as an adhesion layer, and then a 100 nm silver film, were deposited by sputtering. Finally, the platform was coated with a conformal 10 nm SiO_2_ layer, *via* e-beam physical vapour deposition process.

**Figure 1.**
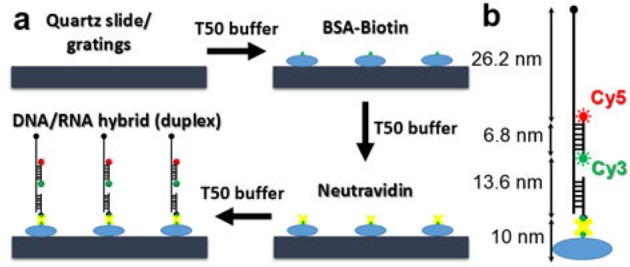
(a) The immobilization process employed for the duplex on the quartz or grating surface; (b) the DNA/RNA duplex molecule used in the SM studies

For SM study, Cy3/Cy5 fluorophore-labelled DNA-RNA (duplex) hybrid molecules were affixed to the nanogap-embedded gratings, and to quartz slides as control samples. The samples were prepared by first dispensing a 50 μL aliquot of 1× T50 buffer solution (10 mM TRIS, 50 mM NaCl, pH 8.0), followed by a 10-minute incubation using 50 μL of 1 μg/μL biotinylated bovine serum albumin (BSA-Biotin) in T50 buffer. Excess BSA-Biotin (not immobilized on the surfaces) was washed away using 50 μL fresh T50 buffer. Neutravidin (50 μL, 1 μg/μL) was then dispensed and incubated for five minutes to allow it to attach to the BSA-Biotin, followed by another T50 buffer rinse to remove excess neutravidin. Finally, the duplex molecules were added with imaging buffer (20 μL 5× T50 buffer, 15 μL of 666.7 mM MgCl_2_, 1.6 μL D-Glucose, 1 μL Gloxy, and 74.2 μL Trolox) to stabilize the fluorophores and avoid any blinking (as shown in Figure 1a). Figure 1b shows the duplex molecule, which contains an RNA backbone (137-base) with biotinylated DNA (29-bp) attached to the substrate, DNA (20-bp) tagged with Cy3 and Cy5 at two ends, and an RNA tail (77-base) extending into the aqueous solution. Considering that the Cy3-Cy5 dye separation (6.8 nm) results in only a ∼20% nominal Förster resonance energy transfer (FRET) efficiency, we can study the fluorescence behaviour of Cy3 and Cy5 individually.

In order to construct the flow cell on the gratings and quartz platforms, a Dremel tool with a 0.75 mm diamond-coated bit was used to drill a set of entry and exit holes for fluid on either side of the glass slide of the flow cell (see Figure S6). An individual grating was secured in the centre of a piece of glass slide using 5 Minute Epoxy (Devcon Home); glass slide strips were then placed alongside the edges of the grating so that the grating was secured between the two holes. The slides and coverslips were first cleaned and then blow-dried by a high-pressure N_2_ flow, and passed over a diffuse flame. Once cooled, double-sided tape was used to separate each set of holes and define the ends; a clean coverslip was then placed on top. Finally, 5 Minute Epoxy was used to close the ends.

### 2.2 Instrumentation and Measurement Details

SM fluorescence measurements were performed on an Olympus IX-71 inverted microscope, with a top prism-based pTIRFM or bottom epifluorescence excitation setup. Excitation sources were 532 nm and 642 nm diode-pumped solid-state lasers (100 mW, Spectra Physics, Excelsior One). The attenuation of laser power to the appropriate fluence was achieved with a 532 nm or 633 nm zero-order half-wave plate (Thor Labs, WPH05M-532/-633), coupled with a broadband polarizing beam splitter (CVI) and neutral density filters (ThorLabs, NE40B, NE30B, NE20B, NE10B, NE06B, NE05B, NE04B, NE03B and NE02B). The emission from the dyes was collected using either a UPlanSApo 100× oil-immersion objective (Olympus, numerical aperture (NA) = 1.40) or a UPlanSApo 60× water-immersion objective (Olympus, NA = 1.20), with the emission being directed toward one of the following filter sets: Filter Set 1: TIRF excitation at 532 nm short-pass filter (Chroma, HQ545lp) for Cy3 channel; Filter Set 2: TIRF excitation at 642 nm short-pass filter (Chroma, zet488/647m, zt488/647rpc) for Cy5 channel; and Filter Set 3: epifluorescence excitation at 532 nm and 642 nm (Chroma, zt532rdc, HQ545Ip). The emission spectra of Cy3 and Cy5 were separated following the excitation filter set using a dichroic mirror (Chroma, 630dcxr), and the image was focused onto an Andor iXon^+^ EMCCD camera, with the same camera gain used for all substrates. The integration time varied for different conditions. The fluorescence signal intensities were calculated after taking into account the final background intensities on the platforms (grating/quartz).

## 3. Results and Discussion

### 3.1 Characterization of Gratings with Embedded Nanogaps

**Figure 2.**
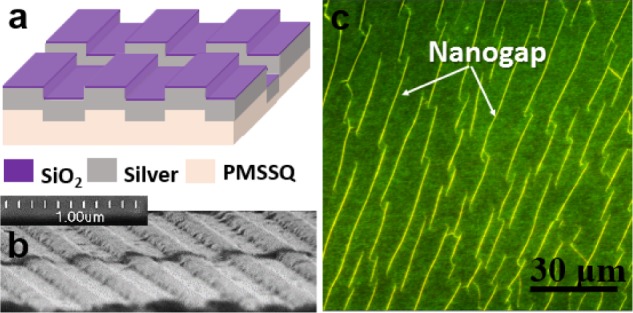
(a) Schematic of a grating with an embedded nanogap; (b) cross-sectional SEM image of the grating substrate, showing the 100 nm Ag gratings with 10 nm SiO_2_ capping layer and an embedded nanogap; (c) fluorescence image (false-colour map) of nanogap-embedded Ag gratings coated with Rhodamine 6G (R6G), using a 40× objective

Figure 2a shows a schematic of the SiO_2_-capped silver plasmonic grating with an embedded nanogap, suitable for SM fluorescence studies. Scanning electron microscopy (SEM) imaging was performed on gratings with nanogaps embedded at the edges of the silver grating substrate, in order to analyse the nanogap cross-sectional areas (Figure 2b). The nanogaps were found to be 20–200 nm wide (see also Figure S1). Fluorescence imaging of a 30 nm dye-doped PMSSQ matrix layer on a grating with embedded nanogaps showed a high density of randomly distributed nanogaps with different lengths and widths (Figure 2c). The variety of nanogap dimensions provides us with a multitude of variables that may affect the LSPR enhancement phenomenon and be directly responsible for the distribution in fluorescence intensities and enhancement factors, as well as the DNA/RNA hybrid (duplex) characteristics seen in the later sections.

### 3.2 SPR Coupling Properties

#### 3.2.1 Dependence of SPR Coupling on Excitation and Emission Angles

**Figure 3.**
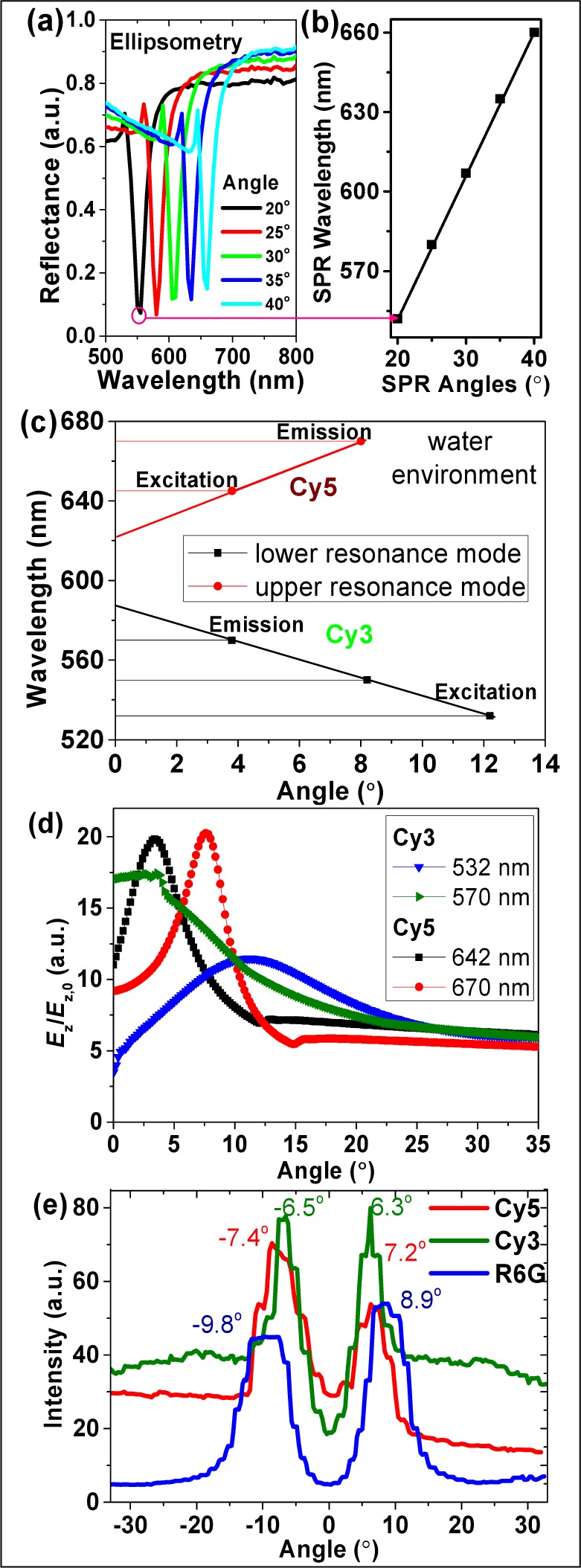
Experimental angle dependence for SiO_2_-capped Ag gratings in an air environment: (a) reflectance at multiple wavelengths and incidence angles; and (b) dispersion curve showing the upper resonance mode of coupling over the visible range. Simulated angle dependence for SiO_2_-capped Ag gratings in an aqueous environment: (c) dispersion curve; (d) max E-field *(E_z_/E_z_*_,0_, i.e., E-field in z direction (normal to grating surface) divided by the incident E-field) for different wavelengths. (e) Fluorescence intensity vs. excitation angle for different dyes spin-coated on the surface of Ag gratings (10 μM concentration, and 30 nm thickness in a PMSSQ matrix, using a 60x water-immersion objective with epifluorescence microscope; laser power for Cy3, Cy5 and R6G is 50 mW, 50 mW and 4 mW, respectively).

SPR is an angle-dependent phenomenon (Equation S1), requiring precise angle matching for a given wavelength to ensure the highest coupling efficiency [16]. Grating SPR coupling characteristics were measured by reflectance as a function of p-polarized light, incident on the grating surface at angles between 20° and 40° as measured from the normal to the substrate plane (Figure 3a, see also Figure S2). For each incidence angle, a specific wavelength range exists over which coupling occurs, leading to sharp dips in reflectance. Figure 3b shows the corresponding upper resonance mode dispersion curve for the gratings, with air as the dielectric. The dispersion curves of the SiO_2_-coated silver gratings in an aqueous environment, used for SM studies, were calculated by finite-difference time-domain (FDTD) simulation (Figure 3c). The angular dependence of both SPR coupling excitation and directed surface plasmon-coupled emission (SPCE) [18,19] determines the angles required in the optics setup to allow optimal fluorescence imaging. The inverted microscope has a variable angle lens with the ability to change the laser incidence angle and, thus, the grating excitation. Fluorophores are first excited by the evanescent SPR field; the SPCE then converts the isotropic emission of the fluorophores within ∼250 nm of the metal surface into directed emission [18,19].

This information can be used to describe how different fluorophores will respond to our grating system. For example, rhodamine 6G (R6G) and Cy3 are excited using a 532 nm green laser, while Cy5 is excited with a 642 nm red laser. The emission is then captured and analysed as the angle of excitation is varied between the extrema of the 60x objective. R6G dye has its excitation peak at 532 nm and its emission peak at 550 nm [25], which couple to our gratings at 12.2° and 8.1°, respectively. Likewise, the SPCE peaks for Cy3 (570 nm) and Cy5 (670 nm) [26] are located at 3.8° and 8°, respectively. As seen in Figure 3d, the maximum value for the E-field (*E*_z_/*E*_z,0_) is achieved at the resonance angles for those wavelengths. Notably, the angle sensitivity of the E-field for various wavelengths is different for this plasmonic grating platform, which is related to the SPR angles as well as the resonance modes.

The emission is always captured at the point where the best coupling takes place (peaks on the graph below). Figure 3e shows the emission from the three dyes, spin-coated within a 30 nm PMSSQ matrix over the silver gratings, as a function of the excitation angle. Each dye has a corresponding region of maximal SPR coupling. Since the power incident over any given angle is kept constant, the main reason for intensity variations is the coupling enhancement [18,19]. The phenomenon that the lowest intensity for Cy3 appears at 0° excitation angle among all angles is consistent with the lowest E-field obtained at 0° under Cy3 excitation wavelength (532 nm), as shown in Figure 3d. Similarly for Cy5, the E-field at 0° excitation is higher than that at large incident angles (> 15°), leading to a higher fluorescence intensity. The intensity, position and width of the fluorescence peaks are also related to the excitation spectra and quantum yields of the fluorophores.

However, the emission side of the angle dependence cannot be optimized or manipulated within our system, due to the fixed objective with this apparatus. Given that the photodetector (camera) capture window is 100×50 μm^2^ for 60x objective and 60×30 μm^2^ for 100x objective, it became clear that Cy3-emitted light could be captured by the detector, while most of the Cy5-emitted light was directed outside the capture area (see Supplementary Figure S3). This phenomenon is the major reason for the loss of Cy5 intensity when performing SM studies using this system.

#### 3.2.2 Dependence of SPR Coupling on Dye-metal Distance

**Figure 4.**
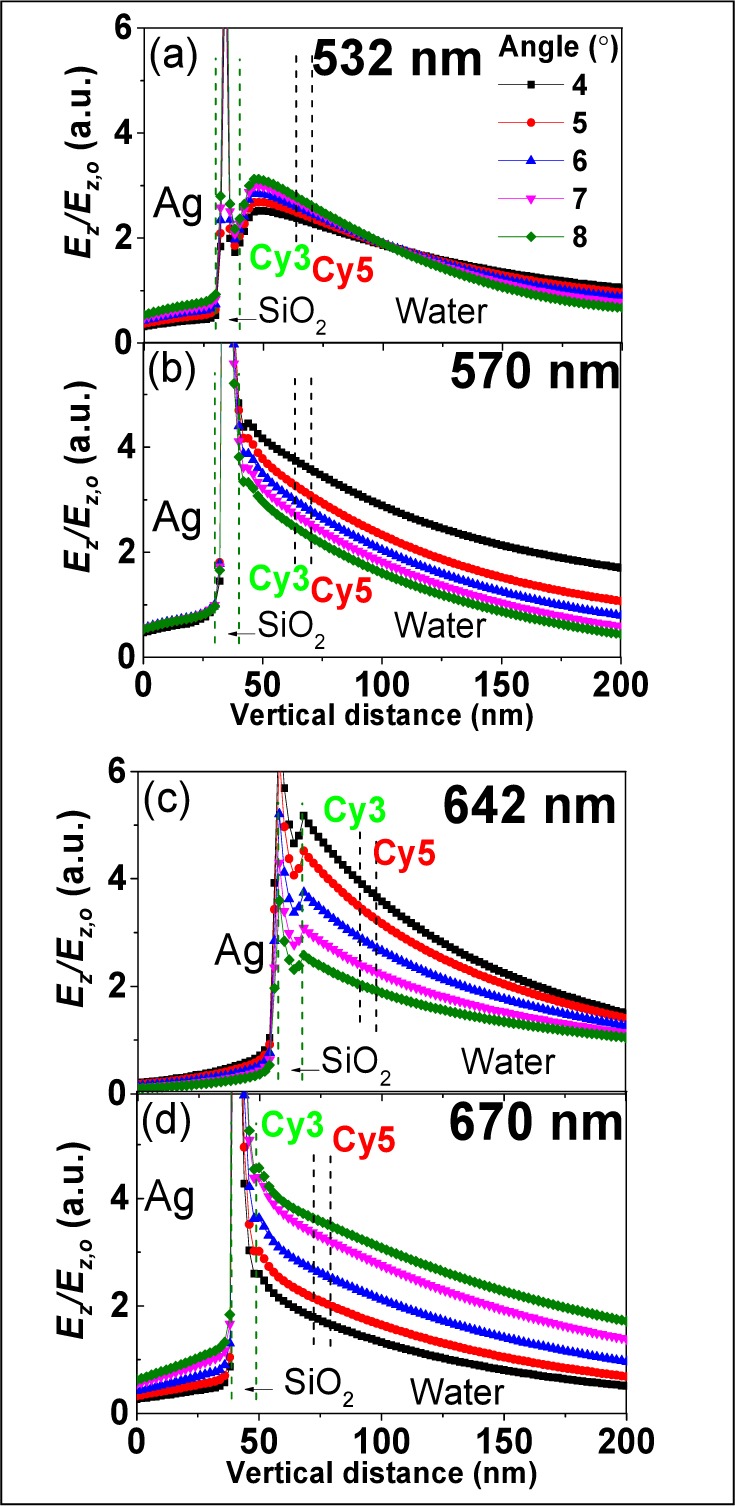
Simulated E-field varying with vertical distance for different angles and wavelengths: (a) 532 nm, (b) 570 nm (c) 642 nm and (d) 670 nm

The distance of dyes from the metal-dielectric interface is also an important metric for enhancement, due to the evanescent E-field created by SPR coupling [27]. E-field values drop to ∼2–4 for dyes located more than 20 nm away from the SiO_2_ surface for all these wavelengths. There is virtually no difference in E-field strength (∼0.2) at Cy3 and Cy5 excitation and emission wavelengths within 6.8 nm vertical distance from the surface (Figure 4), indicating that E-field strength is not a dominant factor for the difference in fluorescent signals between Cy3 and Cy5 under the same excitation conditions (wavelength and angle). However, Cy5 displays a stronger E-field than Cy3 when both dyes are under the optimization of excitation and emission. The E-field for the Cy3 excitation wavelength is less sensitive to incidence angles than other wavelengths, which is consistent with Figure 3d. E-field values varying in vertical distance at different position on the gratings (grooves and peaks), with different incident wavelengths, are discussed in Supplementary Section SI-4 (Figure S4).

### 3.3 SM Fluorescence Studies of Gratings with Randomly Embedded Nanogaps

#### 3.3.1 Comparison of Gratings with Embedded Nanogaps Using Epifluorescence Microscope and TIR-quartz with the Same Laser Power for Cy3

**Figure 5.**
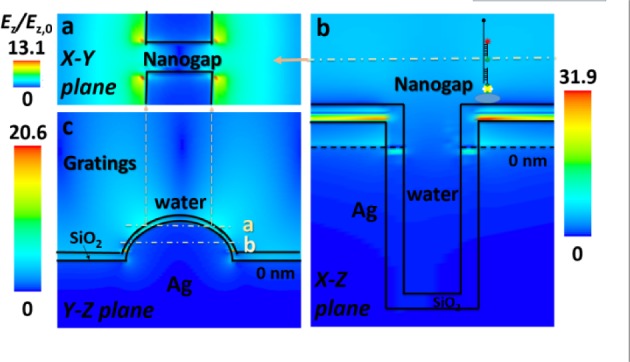
E-field in a 3D simulation of a nanogap-embedded grating, at a 570 nm emission wavelength under a SPCE angle (3.8°) for Cy3: (a) X-Y plane showing where Cy3 dyes may exist, (b) X-Z plane showing maximum E-field in nanogap; and (c) Y-Z plane showing gratings far away from nanogap

SM studies (see Figure S6) were performed by controlling the output power from a 532 nm solid-state laser source through a 60x water immersion objective. Direct comparison of SM imaging between quartz slides and plasmonic gratings in epifluorescence mode was not possible, as quartz fails to provide analysable single-step time traces, even with the highest laser power (75 mW) and camera gain (100), due to the low SNR in epifluorescence mode. The poor SNR in epifluorescence mode is due to far-field illumination that excites the dyes throughout the bulk depth, as opposed to the shallow depth (<100 nm) evanescent field illumination in TIRF mode. This adds the additional noise from the dyes in the solution that are not immobilized on the surface, and reduces the resolvability of the duplex. Meanwhile, the silver SPR gratings generate a strong evanescent E-field at the metal-dielectric interface, improving the SNR even in epifluorescence mode and allowing SM imaging. The nanogaps embedded in the gratings result in LSPR (Figure 5 and Figure S5), which further concentrates the E-field formed by light coupling to the gratings, resulting in much higher local fluorescence enhancement [28,29]. Since the emission intensity enhancement of fluorophores is proportional to the local E-field intensity enhancement (|*E*_z_/*E*_z,0_|^2^), the nanogaps embedded in the gratings are able to achieve a maximum fluorescence enhancement approximate 900-fold according to simulation results. However, the inevitable problems with the experiment (even though we tried to minimize these in this study)—such as light collection, quenching, photobleaching of fluorophores, and the position and configuration of DNA-RNA duplex molecules—could have reduced the experimental enhancement, compared with the simulated values. The highest E-field is located at the upper edge of the silver grating's intersection with the nanogap, where the sudden field discontinuity led to extremely crowded surface charges [22]. Thus, the measurements from the quartz were acquired using prism TIRFM, which represents the gold standard for SM fluorescence imaging, and those from the gratings were acquired through the epifluorescence mode.

**Figure 6.**
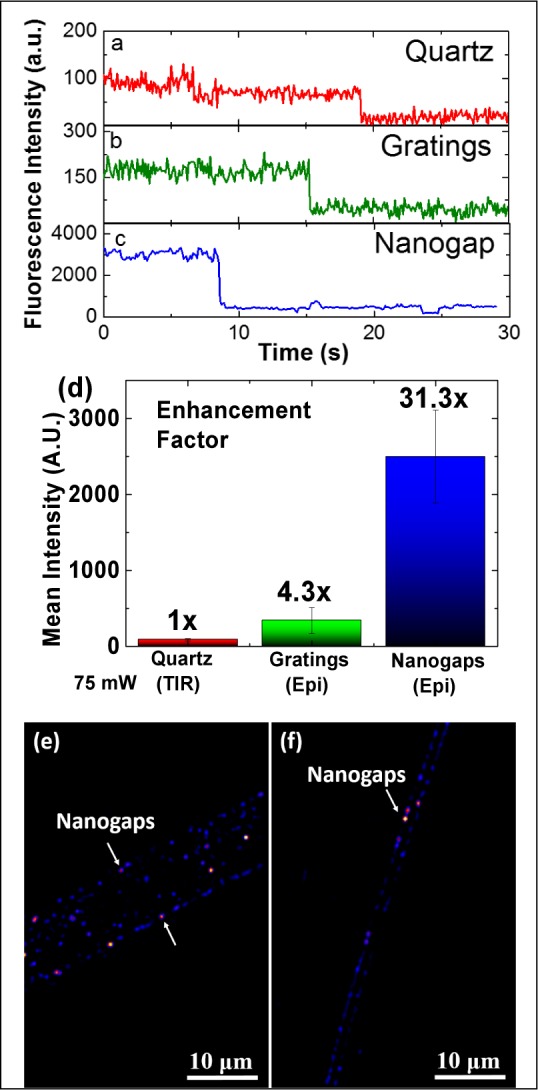
Representative fluorescence time trace of a single Cy3 fluorophore on (a) a plain quartz substrate, (b) a silver grating, and (c) the nanogap-embedded silver grating, using an incident laser power of 75 mW. All time traces are taken using the 60x objective. (d) Fluorescence intensity (with a camera gain of 1) and enhancement factor, for a single Cy3 fluorophore on different substrates. (e-f) Fluorescence images (false-colour maps) for the nanogap-embedded silver grating using the 60x objective. The arrows indicate nanogaps.

A statistical analysis was performed on 45 single molecules extracted from fluorescence movies on different substrates. Single-step photobleaching (Figure 6(a-c)) was seen in both the silver gratings in epifluorescence mode and the quartz in TIRFM mode (TIR-quartz). Interestingly, the intensities recovered from the single molecules on the gratings are routinely higher than those achieved in the TIR-quartz. The bleaching time is also reduced in the presence of metal (i.e., silver), which can be explained by the overall increase in effective radiation in the vicinity of the fluorophore [30]. Figure 6d shows that the mean SM fluorescence enhancement factor for molecules on the gratings and in the nanogaps, with respect to the TIR-quartz intensity, is 4.3-fold and 31.3-fold, respectively. The energy available to the fluorophore, asa result, is effectively increased by a factor of these enhancement factor values. This in turn increases the overall fluorescence intensity and reduces bleaching time. Figure 6(e-f)shows fluorescence images for gratings with randomly sized and distributed nanogaps captured by CCD (more fluorescence images can be seen in Supplementary Figure S7). The propagation of a certain amount of light along nanogaps may create cross-talk, which reduces the detectability of SMs in the nanogaps; however, the extremely high E-field on the edge of the nanogaps (i.e., separate hotspots) leads to much higher fluorescence intensity that increases SNR, resulting in the recognition of SM behaviour in the nanogaps.

#### 3.3.2 Comparison of Three Platforms Using an Epifluorescence Microscope for Cy3

**Figure 7.**
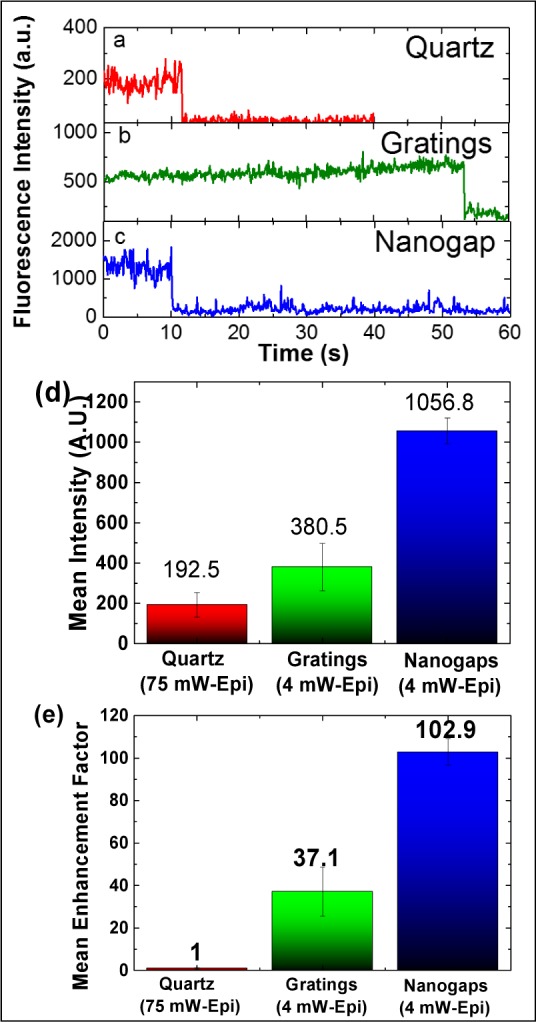
Representative single-Cy3 fluorescence time trace, using a 100× objective in epifluorescence mode, on (a) plain quartz using 75 mW incident laser power; (b) the silver grating using 4 mW incident laser power; and (c) the silver grating with embedded nanogaps using 4 mW incident laser power. (d) Fluorescence intensity with camera gain of 100, and (e) enhancement factor for a single Cy3 fluorophore on different substrates (calculated using a linear relationship between laser power).

Since SM fluorescence behaviour on a plain quartz substrate could not be observed at a lower magnification (60x objective) with an epifluorescence setup, comparative imaging was performed using a higher magnification (100x objective) in epifluorescence mode, to improve the overall SNR for the plain quartz, due to its higher NA and better light collection, as well as its better pixel resolution. Figure 7(a-c) shows the comparative SM behaviour on the three substrates. A laser power of 75 mW is needed for an output intensity of 250 a.u. (100 camera gain) for quartz, while a laser power of 4 mW is able to produce fluorescence intensity of 600 a.u. and 1500 a.u. on gratings and nanogaps, respectively. It became clear that the overall signal enhancement on the gratings and within the nanogaps was so high that using the same laser power as we did for quartz (75 mW) would completely saturate the camera apparatus. Despite the lower power requirements, gratings and nanogaps produce a much higher overall fluorescence intensity when compared to the plain quartz substrates.

The overall reduction in the power of the excitation laser has a direct effect on the bleaching times, as well. The time taken to photobleach increased from 11 s on quartz to 55 s on the gratings. The bleaching time on the nanogaps is closer to that of the quartz substrate, but with the advantage of a 7.5x fluorescence intensity at a ∼19x lower excitation power (Figure 7d) (i.e., the mean nanogap enhancement factor for nanogaps is 102.9x compared with the quartz substrate (Figure 7e)).

#### 3.3.3 Comparison of SM Fluorescence Enhancement Factors between Cy3 and Cy5

**Figure 8.**
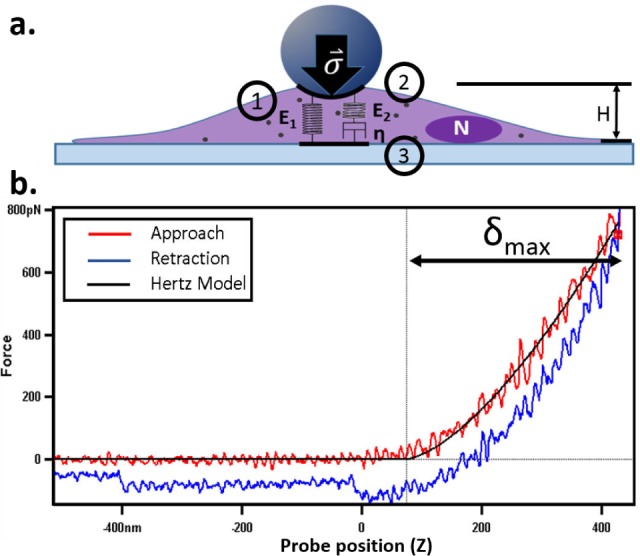
Representative SM fluorescence time trace using a 60× objective in epifluorescence mode on nanogaps: (a) Cy3 and (b) Cy5. Comparison between Cy3 and Cy5: (c) fluorescence intensity with a camera gain of 60, and (d) mean enhancement factors on gratings and nanogaps, with respect to TIR-quartz substrates, using the 60× objective. The laser power on the quartz, and on both gratings and nanogaps, is 75 mW and 4 mW for Cy3, and 50 mW and 4.3 mW for Cy5, respectively.

In order to compare the angle dependence of SPR coupling and SPCE, we analysed the SM fluorescence enhancement factors obtained by exciting Cy3 and Cy5 on the DNA/RNA duplex molecule (Figure 8). Since both Cy3 and Cy5 are present on the same molecule, the enhancement factors are determined experimentally by exciting each dye separately with a green (532 nm) or red (642 nm) laser. The enhancement of Cy3 on gratings is ∼10x, with respect to (wrt) TIR-quartz, and ∼35* in nanogaps. Enhancement values are substantially lower for Cy5, with a grating enhancement factor of 5.5x and a nanogap enhancement of 13.8x (wrt TIR-quartz). The ∼2–3x lower enhancement factor values obtained with Cy5 dye persist across the gratings and nanogap substrates when compared to the Cy3 dye on the same substrates. SPR coupling can improve the quantum yield of dyes, and this effect is more significant for fluorophores with a lower quantum yield [18–20]. Owing to the fact that Cy3 has a quantum yield of 0.15 and Cy5 a quantum yield of 0.28 [31], the quantum yield enhancement for Cy3 is larger than that for Cy5, which is one of the reasons for the differences in the two SM fluorescence enhancement numbers. On the other hand, both dyes were excited with the respective excitation lasers at the appropriate SPR angle to give the maximum enhancement. The reason for such a reduction in enhancement numbers with the Cy5, in comparison to the Cy3, is that the SPCE causes a low capture efficiency for the Cy5 emission, as discussed in the previous section.

## 4. Conclusion

Silver plasmonic gratings with embedded nanogaps were introduced for SM visualization using an epifluorescence microscope. Cy3/Cy5-labelled DNA/RNA hybrid duplexes were affixed to SiO_2_-capped silver gratings, produced through a cost-effective nano-imprint lithography technique using an HD DVD as a mould. The nanogaps locally concentrated the coupling E-field generated by the gratings, which is demonstrated by both experiment and simulation. The SM fluorescence intensity of fluorophores at nanogaps in epifluorescence mode showed more than a 30-fold and 100-fold mean enhancement, compared to those observed on a quartz substrate under TIRFM and epifluorescence microscopy, respectively. The capabilities of fluorescence enhancement of gratings and nanogaps enables a reduction in the required laser power, resulting in an increased photobleaching time from 11 s on quartz to 55 s on gratings. Enhancing the Cy5 signal by reducing its SPCE angle requires the proper tuning of grating parameters, including the refractive index and thickness of dielectrics. These simple gratings with embedded nanogaps provide a low-cost approach to the performance of SM studies by enabling the utilization of an epifluorescence apparatus, instead of the more expensive TIRFM or confocal microscopy setup.
